# Reconfigurable switching between reflecting/absorbing modes in VO_2_ assisted graphene-coated hemispherical dielectric hole arrays

**DOI:** 10.1038/s41598-022-11476-2

**Published:** 2022-05-12

**Authors:** Shiva Hayati Raad

**Affiliations:** Department of Electrical and Computer Engineering, Tarbiat Modares University, Tehran, Iran

**Keywords:** Applied optics, Optoelectronic devices and components

## Abstract

In this paper, a graphene-coated dielectric hole array is used to design a reconfigurable switchable optical reflector/absorber device. The design benefits from the collective excitation of localized surface plasmon resonances of graphene-coated hole array, providing simpler fabrication fellow and more compact structure with respect to graphene-coated spherical nanoparticle array with similar plasmonic behavior. Geometrical parametric study of the reflecting mode shows that the device has lots of degrees of freedom for spectrum tuning and can highly tolerate fabrication imperfections. Moreover, the reflection rate is slightly affected by the dielectric substrate height, which can be tuned to achieve strong absorption by backing it with a metallic mirror. The designed absorber efficiently captures a wide range of obliquely incident transverse electric (TE) and transverse magnetic (TM) waves. Also, the operating frequency of both reflecting and absorbing modes can be tuned after fabrication, thanks to the two-dimensional nature of graphene material. Finally, using vanadium dioxide (VO_2_) phase change material, the switchable reflector\absorber mode of the device is also exhibited.

## Introduction

Graphene-coated dielectric or metallic spherical nanoparticles has been used in the design of different optical devices respectively due to the localized surface plasmon excitation arising from the charge carriers in the graphene shell and the interplay between plasmons supported by the bulk core and the graphene shell^1^. For instance, the near field enhancement provided by the spherical graphene shells is discussed in the framework of modified Mie Lorenz theory and by extracting equivalent RLC ladder circuit for the resonance behavior of the plasmonic nanoparticles^2^. Furthermore, graphene oxide wrapped gold nanoparticles are utilized for intracellular Raman imaging and drug delivery^3^. In other applications, the localized surface plasmon resonances of the graphene wrapped nanoparticles are used to design electromagnetic cloaks and superscatterers^4,5^. Moreover, reconfigurable sub-wavelength strong absorbers are proposed by square/hexagonal lattice of graphene shells with spherical morphology^6–8^. Graphene hollow spheres have also found applications in supercapacitors, being superior to their planar counterparts^9^. Also, graphene-coated spherical shells are proposed as a cover for cylindrical wires to reach dual-polarized extinction cross-section enhancement^10^. Although fabrication of graphene-based spherical geometries is feasible considering current nanofabrication technology^11^, still, they suffer from complex fabrication fellow. Thus, proposing a geometry with similar optical performance, yet a simpler fabrication process, is helpful for practical usage.

Previous studies show that extraordinary transmission through arrays of sub-wavelength holes in thin metal films is due to the excitation of surface plasmons^12^. Generally, graphene-based structures provide enhanced optical performance with respect to their metallic and high index dielectric counterparts because of being low-loss and supporting highly confined waves^13^. Thus, the present research aims to investigate the graphene-coated hole arrays as an alternative plasmonic device to metallic hole arrays, benefiting from the exotic properties of graphene material to reach a switchable reflector/absorber performance. Metallic hole arrays support pronounced Fano resonances as interference between light directly transmitted through the holes and light indirectly scattered because of the excitation of localized surface plasmon polaritons, propagating surface plasmon polaritons, or/and waves due to Wood’s anomaly^14^. A potential application of the metallic nano-hole array is the refractive index sensing for label-free detection^15^ and can be fabricated by low-cost colloidal sphere lithography^16^.

By the use of hole arrays in conjunction with other plasmonic elements or material combinations, the optical performance can be further manipulated. For instance, strong coupling between a nanoparticle dark mode with a high-order metallic hemispherical nano-cavity, bright mode has been exploited for optical field enhancement through Fano resonance excitation^17^. Also, absorption enhancement in the graphene sheet is obtained by a square lattice of air holes in the high index dielectric^18^. Moreover, graphene/hBN/graphene multilayer hole array leads to a photonic plasmonic absorption of the electromagnetic wave^19^. The present paper benefits from the combined use of graphene-based hole arrays and phase change materials to design a reconfigurable switchable reflector/absorber device. Gate tunable feature of graphene material and thermal tunable property of the VO_2_ material provide two degrees of freedom for the dynamic response achievement^20^. A switchable absorber/reflector device provides a wide range of applications such as active camouflaging, modulating, electro-optic switching, antenna beam steering, hyper-spectral imaging, and compressive imaging^21,22^. The paper is organized as follows. The reflecting mode of the square array of graphene-coated dielectric hole array is investigated by exhibiting the excited localized surface plasmon resonances on graphene shells. Later, by using an optical mirror, the reflecting mode is converted to the absorbing mode and its robust performance in terms of the incident angle of the incoming wave is discussed. Finally, a phase change material is used to switch between these modes.

## Results and discussions

In this section, the design of optical reflector, absorber, and switchable reflector/absorber devices using graphene-coated hole arrays are considered. The performance of all devices relies on the excitation of localized surface plasmon resonances on the spherical shell-shaped graphene holes, where the functionality can be controlled by the substrate.

### Reflecting mode

**Figure 1:**
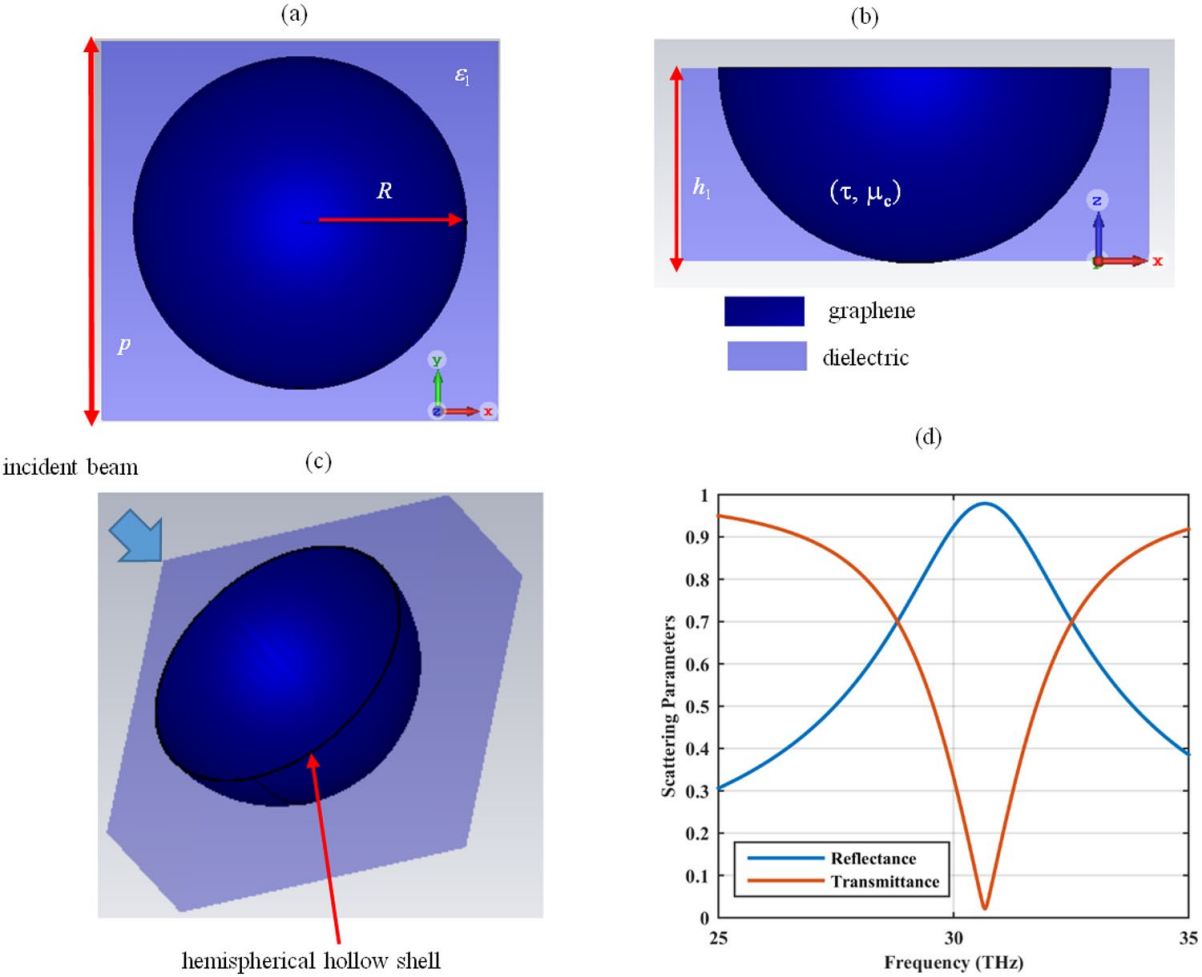
(**a**) Top, (**b**) side and (**c**) three-dimensional views of the proposed unit cell designed using the graphene-coated dielectric hole array (**d**) its reflectance and transmittance. Note that the graphene sheet is wrapped around the sidewalls of the holes. The parameters are as follows: *R* = 100 nm, *ε*_1_ = 2, *h*_1_ = 100 nm, *τ* = 2 ps, and *µ*_*c*_ = 1.5 eV.

Let us consider a graphene-coated hemispherical hole array constructed by the holes with the radius of *R*, embedded in a dielectric medium with the relative permittivity of *ε*_1_, as in Fig. 1. The holes are arranged with the periodicity *p* and are drilled on a substrate with the height of *h*_1_. Two-dimensional graphene material is wrapped around the sidewalls of the hole as hemispherical shells and it is characterized by its optical parameters, namely, relaxation time *τ* and chemical potential *µ*_*c*_. These parameters essentially manipulate the graphene surface conductivity *σ*. Note that there are two approaches to simulate the graphene material for optical applications, either to model it with a zero-thickness shell with the surface conductivity calculated by the Kubo formulas or consider it as a very thin dielectric layer with the thickness *δ* with the equivalent bulk permittivity of ε = 1 + jσ/ωε_0_δ, where the real part of the permittivity is negative. To avoid three-dimensional fine volumetric meshing due to the contrast between the graphene layer thickness and other dimensions, the former approach is applied in the simulations^4,23–25^. The initial parameters are as follows: *R* = 100 nm, *ε*_1_ = 2, *p* = 240 nm, *h*_1_ = 100 nm, *τ* = 2 ps, and *µ*_*c*_ = 1.5 eV. The device is excited from its top side (Fig. 1c) with the first two dominant TE and TM Floquet modes. The reflectance and transmittance, shown in Fig. 1d, confirm that the device can reflect more than 97% of the incoming wave and can be potentially used in the optical reflector design. The electrical size of the device is 0.01 *λ*, confirming its deep sub-wavelength nature. Note that optical reflectors are commonly designed by all-dielectric metamaterials because of their very low dissipation losses^26^. The use of metallic components in the reflector design in the infrared regime is primarily limited due to their ohmic losses and fabrication complexity^27–29^. Thus, using the graphene material, plasmonic reflectors at infrared frequencies can be obtained.

**Figure 2:**
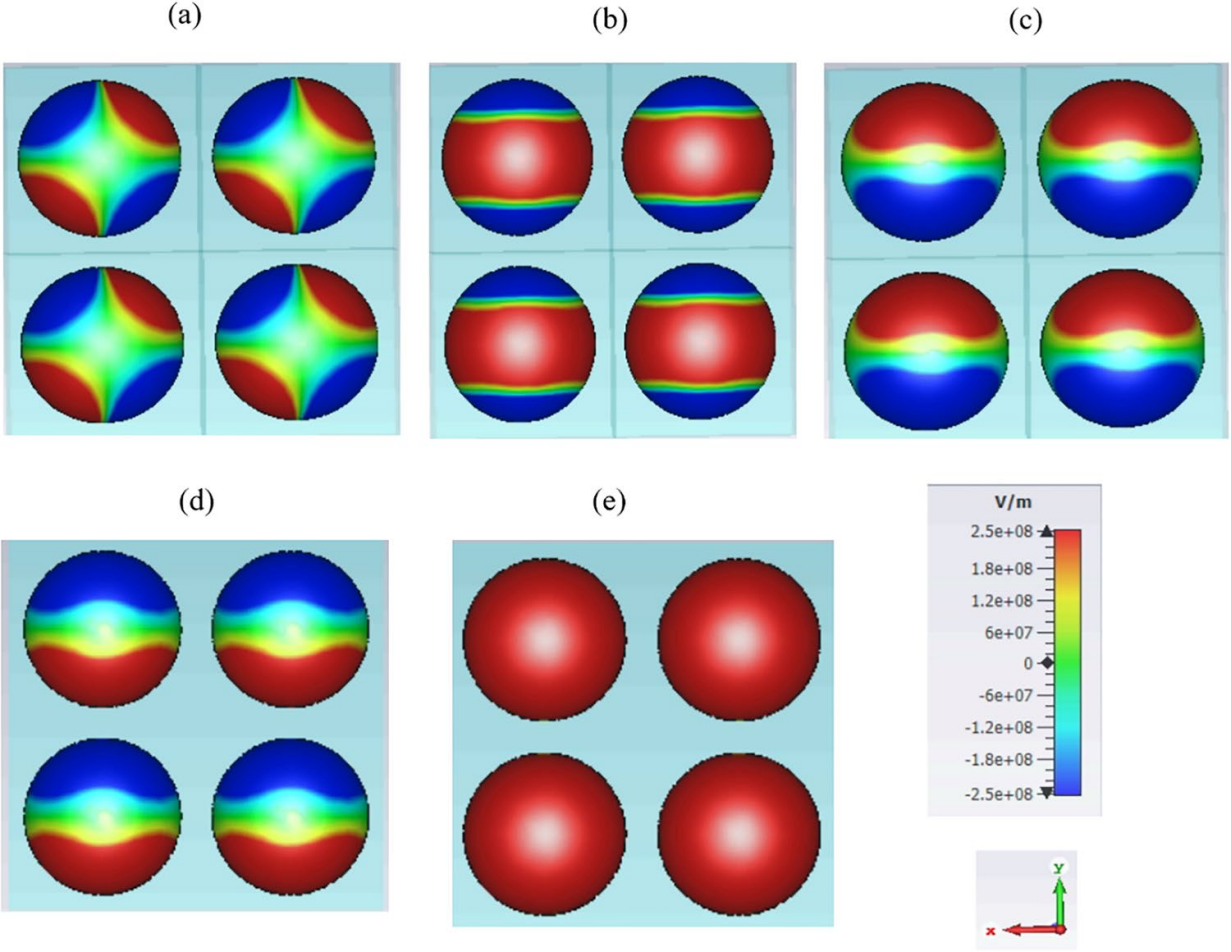
Spatial distribution of the electric field for the structure in Fig. 1 at the reflection peak (30.62 THz) (**a**) *E*_*x*_, (**b**) *E*_*y*_, (**c**) *E*_*z*_, (**d**) normal component and (**e**) tangential component.

To understand the origin of high reflection, in Fig. 2a–c, different components of the spatial electric field are illustrated at the reflection maxima at 30.62 THz. For clear illustration, the backside of the hole is shown. The field distribution shows the excitation of localized surface plasmon resonances with different orders and degrees. Specifically, in Fig. 2a, b and Fig. 2c the quadrupole and dipole plasmonic resonances are respectively observed. Considering a full spherical graphene shell array, the field components illustrate quadrupole modes with various degrees for all spatial components^30^, and here due to the geometrical cropping, the *E*_*z*_ field component has turned to dipole-like mode. The total field is the superposition of the illustrated three orthogonal components in Fig. 2a–c and as Fig. 2d shows, its normal component is a dipole mode^31^. Similar field distributions are observed in graphene-coated spherical particle arrays^7,32^. Importantly, the excited plasmonic resonances in the hole arrays are essentially the same as their particle counterparts, related to each other by Babinet’s principle^15^. Note that due to the homogeneous nature of the structures, the optical response may not differ by exciting the hemisphere from the concave side or converge side. Also, considering the hemispherical configuration instead of a spherical one leads to a compact device while embedding the hemispheres in a dielectric background is fabrication friendly. It is essential to note that graphene surface plasmons can propagate on the curved substrates under full or partial coverage of the surface with graphene^33,34^. Thus, by considering the graphene-coated hole as a partially covered hollow spherical particle, the plasmonic excitation can be further understood. The device can be realized by the combined use of the fabrication method of drilled holes and the graphene coating method. Different approaches such as self-assembly lithography and colloidal sphere lithography have been used for the hole array fabrication^12,16,35^. Moreover, both physical methods (like tape-assist transfer and spin-coating), and chemical methods have been used for wrapping graphene sheets around curved objects^3,11,15,36^.

**Figure 3:**
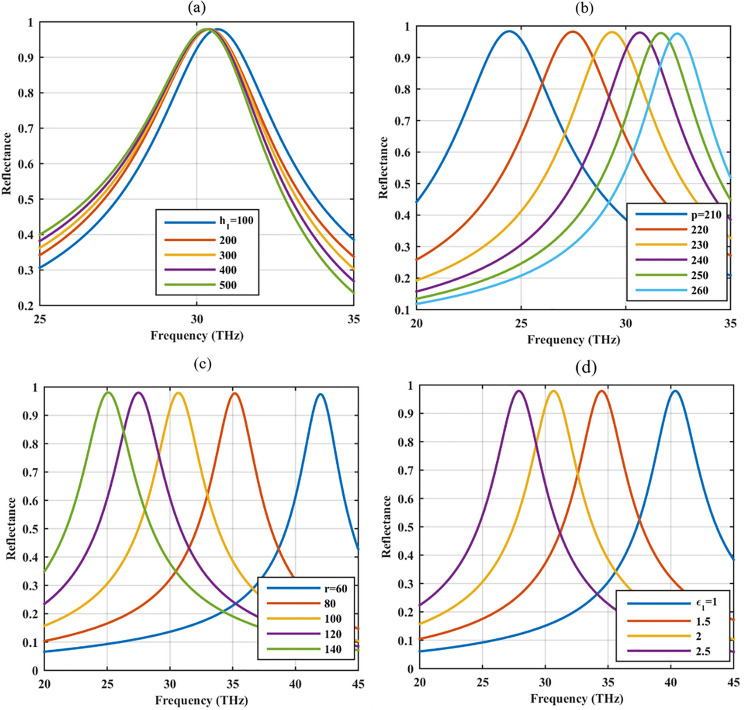
Reflectance of the graphene-coated hole array for different geometrical and material parameters (**a**) substrate height *h*_1_ (nm), (**b**) periodicity *p* (nm), (**c**) hole radius *R* (nm) and (**d**) substrate material permittivity *ε*_1_.

Once the advantage of the graphene-coated hole array with respect to graphene-coated spherical nano-particle array is discussed, it is illustrative to investigate the optical performance manipulation regarding different parameters. In Fig. 3a, the substrate height is varied and it is observed that it slightly impacts the operating frequency and reflection rate. This feature is expected since the optical performance relies on the plasmonic resonances of graphene shells and the low index dielectric substrate heights are still sub-wavelength to be able to cause considerable impact. This feature offers the opportunity to further manipulate the optical resonance to convert the device to an absorber or reflector/absorber, as discussed in the next two sections. In Fig. 3b, the impact of array periodicity on the optical performance is studied. Since plasmonic coupling is stronger in dense arrays, they provide more reflectivity, yet, the amount of reflection is not highly affected by the inter-element distance. Included in Fig. 3, is the influence of the hole radius and material on the optical response. Specifically, Fig. 3c shows that the amount of reflectivity is not affected by the hole radius. The operating spectrum can be manipulated by using different core radii. Similar to the graphene-coated spherical particle array, increasing the hole radius redshifts the operating frequency^32^. When extracting these results, the distance between the adjacent elements is considered 2R+40 nm in all cases. Moreover, Fig. 3d shows that a wide range of dielectric materials can be used to design the graphene-based hole array reflector since the permittivity of the material merely affects the operating frequency. By using higher substrate permittivity, more compact devices can be achieved. Considering all the subfigures together, it can be realized that the geometrical and material parameters do not affect the amount of reflection from this device, yet provide the opportunity for spectrum tuning. This feature is of great importance from the practical point of view since fabrication imperfections can be tolerated.

**Figure 4:**
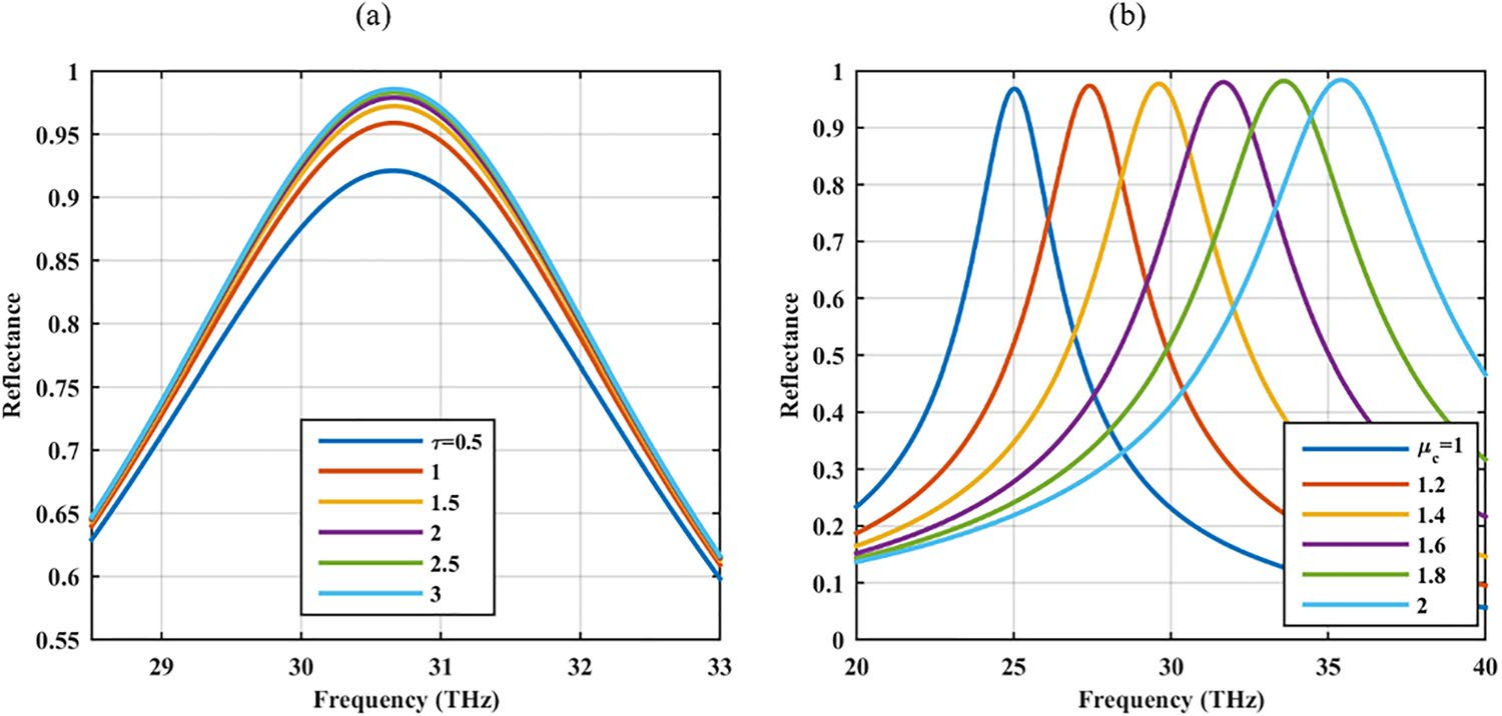
Reflectance of the graphene-coated hole array for different graphene optical parameters (**a**) relaxation time *τ* (ps) and (**b**) chemical potential *µ*_*c*_ (eV).

In Fig. 4, the possibility of optical reflection control using graphene optical parameters is considered. Specifically, in Fig. 4a the relaxation time of graphene material is varied and it shows that once the quality of the graphene material is increased, the optical reflection is also increased. Thus, using high-quality graphene material is essential to reach near-complete reflection. Moreover, Fig. 4b confirms that by manipulating graphene's chemical potential, the operating spectrum can be engineered. The chemical potential of graphene material can be tuned after fabrication by changing the applied bias voltage. Note that the electrical connection of the elements can be provided by thin strips, without affecting the overall response^37^.

### Absorbing mode

**Figure 5:**
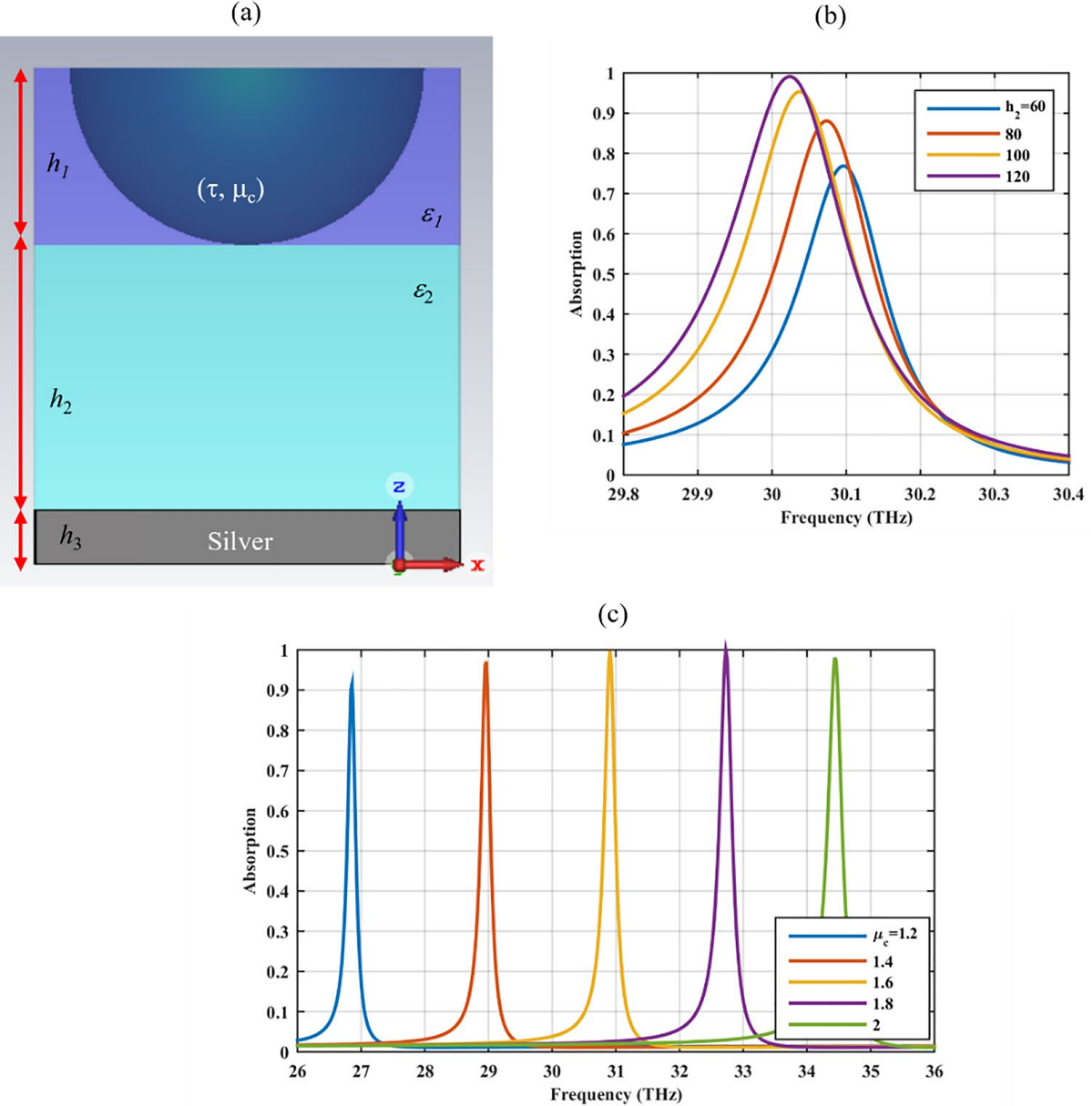
(**a**) Graphene coated hole array in Fig. 1 on top of a reflecting mirror formed by a dielectric with the relative permittivity *ε*_2_ and height *h*_2_, backed by a silver layer and its absorption rate for the (**b**) different substrate heights *h*_2_ (nm) and (**c**) chemical potentials µ_c_ (eV).

In this section, the excited localized surface plasmon resonances in the graphene-based hole array are used to design an optical absorber. The absorption rate is calculated from the *S* parameters^38^. To this end, the hole array has resided on top of a metal-backed dielectric substrate for transmission blockage and its height is modulated such that a perfect absorption is achieved^39^. The metal termination is formed by *h*_3_ = 20 nm silver layer with the surface conductivity of σ_s_ = 6.3 × 10^7^ S/m and it will be replaced by a VO_2_ layer in the metallic phase in the next section^40^. For simplicity, the dielectric material is the same as the one used to reside the hole array previously (*ε*_1_ = *ε*_2_). The final structure is shown in Fig. 5a. By the optimized height of *h*_2_ = 120 nm, perfect absorption is achieved and it is illustrated in Fig. 5b. Moreover, the gate tunable feature of the absorber is illustrated in Fig. 5c, where the absorption rate of above 90% is achieved for the chemical potentials greater than µ_c_ = 1.2 eV.

**Figure 6:**
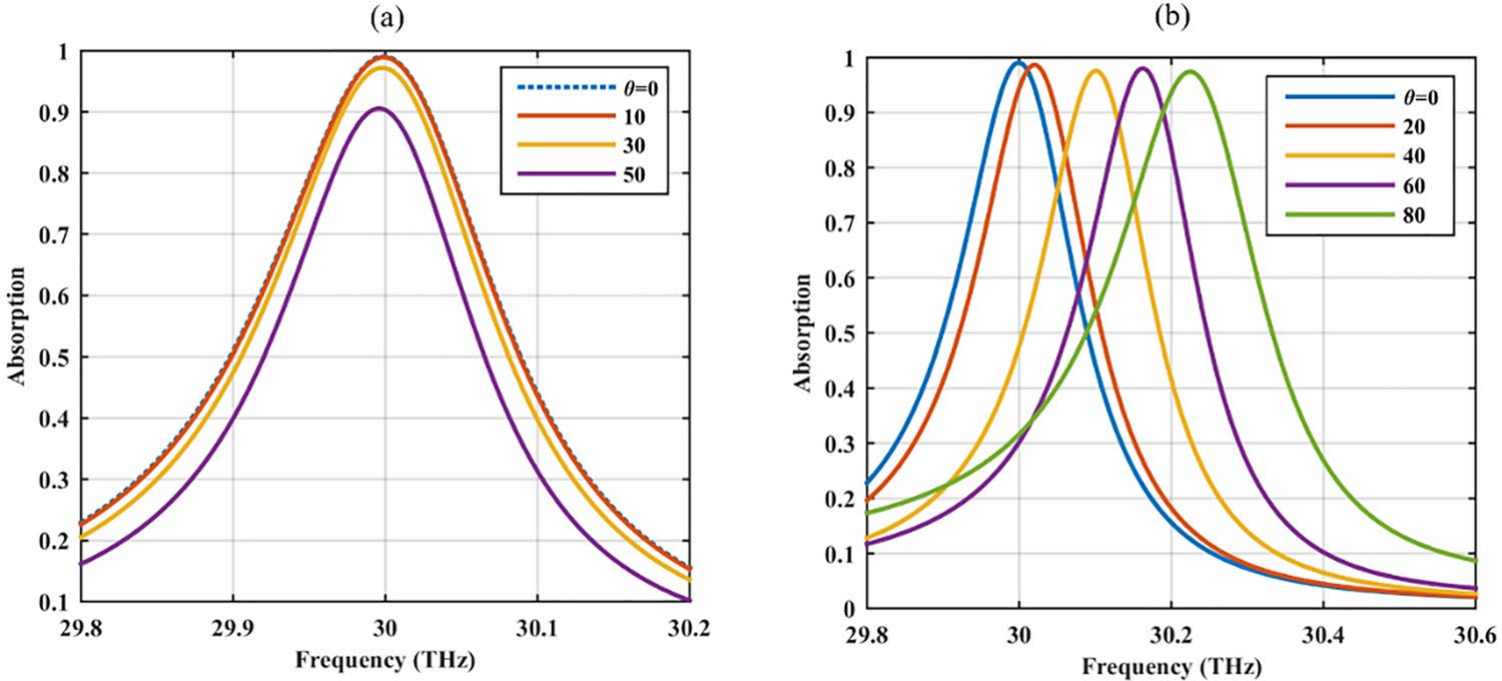
The optical response of the absorber in Fig. 5 for different incident angles (**a**) TE waves and (**b**) TM waves.

The sensitivity of the optical absorption to the incident angle of the incoming wave is investigated in Fig. 6. As Fig. 6a confirms, it is feasible to achieve the absorption rate above 90% for the oblique TE incident waves up to the angle of 50 degrees. Under TM illumination, the absorption is near perfect for any incident angle.

### Switching mode

**Figure 7:**
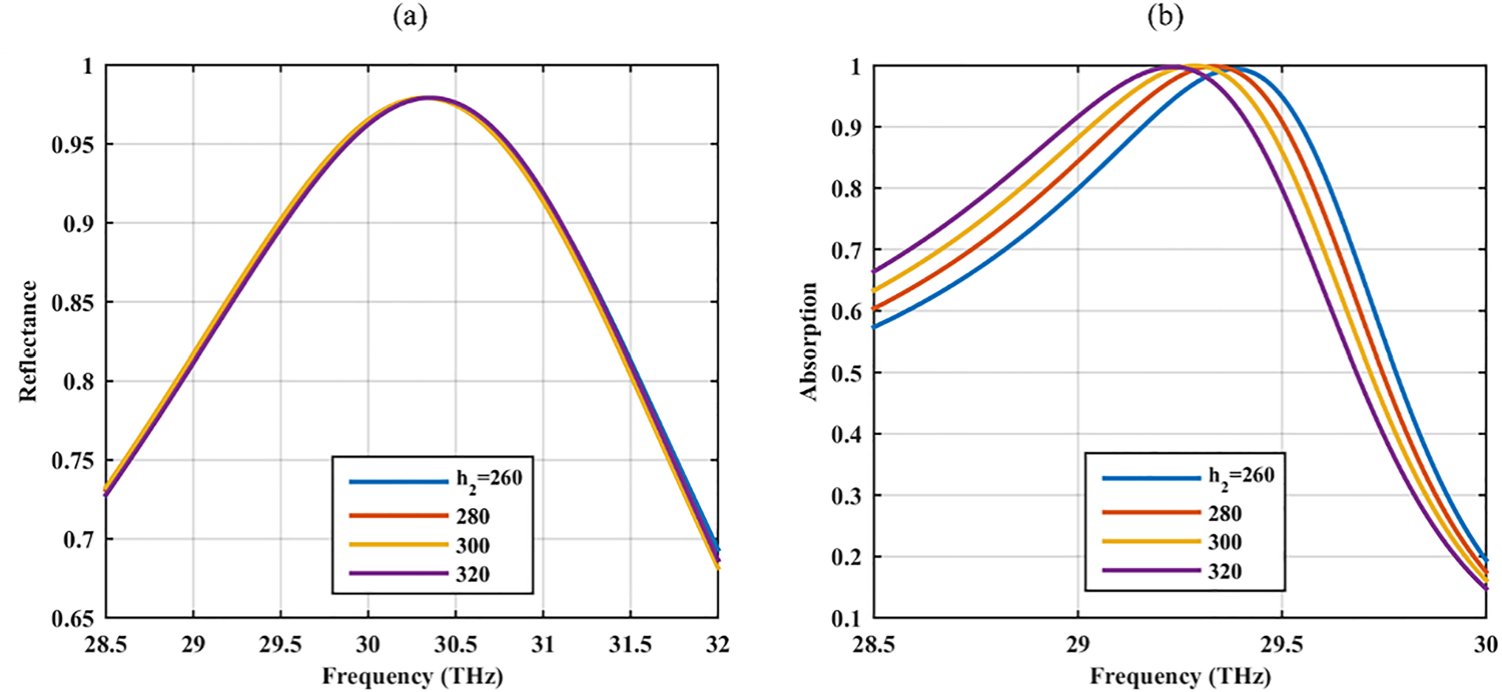
The optical (**a**) reflectance and (**b**) absorption of the proposed device respectively in the insulator and metallic phases of the VO_2_ material for different *h*_2_.

In other to enable switching between the reflecting and absorbing modes, the metallic mirror beneath the proposed absorber is replaced by a VO_2_ layer with the thickness of 10 nm. Due to the phase transition in VO_2_, it behaves as a metal with the surface conductivity of 2 × 10^5^ S/m at the temperature of *T* = 350 K in the metallic phase while behaves as a dielectric with the permittivity of 9 and surface conductivity of 200 S/m in its dielectric phase^41–44^. The choice of thickness is to ensure the transmission blockage in the absorbing mode and the substrate height *h*_2_ ranges from 260–320 nm. Figure 7 shows the optical performance in these two states. Reflectance above 97% and near-perfect absorption are respectively attained in the dielectric and metallic phases of VO_2_ around the same frequency. Importantly, the device has a robust performance against the substrate height.

## Conclusions

Graphene-coated hole arrays are a promising candidate for the future optical device design since they support localized surface plasmon resonances similar to the graphene-coated spherical nanoparticle arrays, yet provide simple fabrication fellow. This geometry can be used either in the reflecting or absorbing mode, originating from the fact the reflection rate is not sensitive to the substrate height, thus can be tuned to achieve a high absorption rate. Interestingly, switching between these two modes is also feasible using a phase change material. The proposed geometry has lots of degrees of freedom for spectrum engineering. Importantly, the performance is reconfigurable due to the use of two-dimensional graphene material.

## Methods

To investigate the optical performance, numerical simulations are conducted in CST commercial software under unit cell boundary conditions and Floquet port excitation. Graphene material has been opted from the software library^45^. The absorption rate (*A*) of the structure is calculated as^46^:

1 $$A = 1 - T - R = 1 - \left| {S_{21} } \right|^{2} - \left| {S_{11} } \right|^{2}$$

where *S*_11_ and *S*_12_ are the scattering parameters.

## Data Availability

The datasets used and/or analysed during the current study available from the corresponding author on request.
